# A Closer Look on Nuclear Radiation Shielding Properties of Eu^3+^ Doped Heavy Metal Oxide Glasses: Impact of Al_2_O_3_/PbO Substitution

**DOI:** 10.3390/ma14185334

**Published:** 2021-09-16

**Authors:** Ghada ALMisned, Huseyin O. Tekin, Antoaneta Ene, Shams A. M. Issa, Gokhan Kilic, Hesham M. H. Zakaly

**Affiliations:** 1 Department of Physics, College of Science, Princess Nourah Bint Abdulrahman University, Riyadh 11564, Saudi Arabia; gaalmisned@pnu.edu.sa; 2 Medical Diagnostic Imaging Department, College of Health Sciences, University of Sharjah, Sharjah 27272, United Arab Emirates; htekin@sharjah.ac.ae; 3 Medical Radiation Research Center (USMERA), Uskudar University, Istanbul 34672, Turkey; 4 INPOLDE Research Center, Department of Chemistry, Physics and Environment, Faculty of Sciences and Environment, Dunarea de Jos University of Galati, 47 Domneasca Street, 800008 Galati, Romania; 5 Physics Department, Faculty of Science, Al-Azhar University, Assiut 71524, Egypt; sh_issa@ut.edu.sa; 6 Physics Department, Faculty of Science, University of Tabuk, Tabuk 47512, Saudi Arabia; 7 Department of Physics, Faculty of Science and Letters, Eskisehir Osmangazi University, Eskisehir 26040, Turkey; gkilic@ogu.edu.tr; 8 Institute of Physics and Technology, Ural Federal University, Ekaterinburg 620002, Russia

**Keywords:** heavy metal oxide glasses, Eu_2_O_3_, Phy-X/PSD, radiation shielding, Al_2_O_3_

## Abstract

In this study, a group of heavy metal oxide glasses with a nominal composition of 55B_2_O_3_ + 19.5TeO_2_ + 10K_2_O + (15−x) PbO + xAl_2_O_3_ + 0.5Eu_2_O_3_ (where x = 0, 2.5, 5, 7.5, 10, 12.5, and 15 in wt.%) were investigated in terms of their nuclear radiation shielding properties. These glasses containing lanthanide-doped heavy metal oxide were envisioned to yield valuable results in respect to radiation shielding, and thus a detailed investigation was carried out; the obtained results were compared with traditional and new generation shields. Advanced simulation and theoretical methods have been utilized in a wide range of energy regions. Our results showed that the AL0.0 sample with the highest PbO contribution had superior shielding properties in the entire energy range. The effective removal of cross-sections for fast neutrons (Σ_R_) was also examined. The results indicated that AL5.0 had the greatest value. While increasing the concentration of Al_2_O_3_ in samples had a negative effect on the radiation shielding characteristics, it can be concluded that using PbO in the Eu^3+^ doped heavy metal oxide glasses could be a useful tool to keep gamma-ray shielding properties at a maximum level.

## 1. Introduction

Even after prolonged exposure to high doses of radiation, many of the glass types retain good internal transmission and exceptional internal quality in terms of bubbles and inclusions. Additionally, some special glasses may tolerate very high cumulative radiation levels without the shield failing [1,2]. However, glass materials have a very wide area in terms of structural concept. The objective and application area also contribute significantly to fulfilling the researchers’ needs by defining the characteristics to be sought in the proposed glass structure. Glasses enriched by trivalent rare-earth (RE) ions are widely utilized in the fabrication of many photonic devices, including lasers, color displays, sensors, LEDs, and optical-fibers used in communication systems [3,4,5,6,7,8]. Glass is an excellent host medium for rare earth (RE) ions, luminescence species effective for a range of photonic applications [9,10], due to its inhomogeneous broadening, low production cost, increased thermal stability, ease of manufacturing, and simplicity of shape. On the other hand, RE ions may be utilized to enhance the luminescence characteristics of heavy metal oxide (HMO) glasses [11,12,13,14].

In contrast to phosphate, borate, and silicate glasses, HMO glasses are better photonic tools due to their broader transparency interval covering the visible to the mid-infrared range, enhanced non-linear optical properties, greater solubility of rare-earth ions, and lower phonon energies. Heavy metal oxide glasses have superb optical and electrical characteristics, including a high refractive index and dielectric constant, which indicate excellent thermal, mechanical, and chemical resistance. On the other hand, much research on the applicability of HMO glasses as radiation shielding materials has been conducted [15,16,17,18]. Although heavy metal oxide glasses are promising candidates for a number of optoelectronic applications, the material density (g/cm^3^) of a glass shield is a fundamental need for possible gamma radiation shielding substances. A material’s atomic number is a property that directly affects its density (g/cm^3^) and the number of electrons in its orbit. The number of electrons in the orbits of elements with large atomic numbers is anticipated to increase as well. When ionizing X-rays and gamma rays interact with materials of high density and atomic number, more consecutive interactions and energy losses occur [19]. These materials allow the ionizing X-ray and gamma ray to lose more energy per unit distance and therefore facilitate the absorption process. As a result of their high material densities, HMO glasses are of great interest to researchers for their nuclear radiation shielding capabilities. In this study, several promising and fairly dense Eu_2_O_3_ reinforced HMO glasses [20] were chosen for this research from the literature. In their research, Pravinraj et al. studied some enhancement strategies on Eu_2_O_3_ reinforced HMO glasses using a regular substitution between Al_2_O_3_ and PbO. Using analytical and modeling techniques, we investigated some of the major contributions of the Al_2_O_3_ additive to the fast neutron and gamma-ray attenuation properties of HMO glasses. The findings will be presented in the context of each glass sample’s Al_2_O_3_/PbO substitution. We hypothesized that substituting Al_2_O_3_ for PbO would alter the gamma-ray and the fast neutron characteristics of HMO glasses. As a result, each observation made throughout the research will be related to a certain scientific theory. Given the prominence of HMO glasses in the literature and their shielding uses in a variety of types of radiation facilities, the present study’s results may provide some interesting and helpful insights into the existing HMO glass and radiation shielding literature.

## 2. Materials and Methods

A series of heavy metal oxide glasses with a nominal-composition of 55B_2_O_3_ + 19.5TeO_2_ + 10K_2_O + (15 − x) PbO + xAl_2_O_3_ + 0.5Eu_2_O_3_ (where x = 0, 2.5, 5, 7.5, 10, 12.5, and 15 in wt.%) were chosen from the literature [20] where the authors conducted a characterization assessment on those glasses’ luminescence capabilities. Their results prompted us to expand the scope of the characterization by including some important glass properties, notably nuclear radiation shielding capabilities. The following table summarizes the compositions of the glass samples examined.

- 55B_2_O_3_ + 19.5TeO_2_ + 10K_2_O + 15PbO + 0Al_2_O_3_ + 0.5Eu_2_O_3_
- 55B_2_O_3_ + 19.5TeO_2_ + 10K_2_O + 12.5PbO + 2.5Al_2_O_3_ + 0.5Eu_2_O_3_
- 55B_2_O_3_ + 19.5TeO_2_ + 10K_2_O + 10PbO + 5Al_2_O_3_ + 0.5Eu_2_O_3_
- 55B_2_O_3_ + 19.5TeO_2_ + 10K_2_O + 7.5PbO + 7.5Al_2_O_3_ + 0.5Eu_2_O_3_
- 55B_2_O_3_ + 19.5TeO_2_ + 10K_2_O + 5PbO + 10Al_2_O_3_ + 0.5Eu_2_O_3_
- 55B_2_O_3_ + 19.5TeO_2_ + 10K_2_O + 2.5PbO + 12.5Al_2_O_3_ + 0.5Eu_2_O_3_
- 55B_2_O_3_ + 19.5TeO_2_ + 10K_2_O + 0PbO + 15Al_2_O_3_ + 0.5Eu_2_O_3_

As a result, we utilized two distinct methods to ascertain the essential gamma-ray shielding characteristics as well as certain mathematical applications to find the effective removal cross-section (R) values for fast neutrons. The glasses’ linear attenuation coefficients were calculated using the general-purpose Monte Carlo program MCNPX (v.2.7.0, Radiation Shielding Information Center, Oak Ridge National Laboratory, Oak Ridge, Tennessee (United States); Advanced Accelerator Applications Los Alamos National Laboratory, Los Alamos, New Mexico (United States)) [21] and the online computing platform Phy-X/PSD(MCNPX 2.4.0) [22]. The aim of this double-tool methodology was to check the appropriateness of the Monte Carlo simulation results.

### 2.1. Simulation Phase of Shielding Parameters

**Figure 1:**
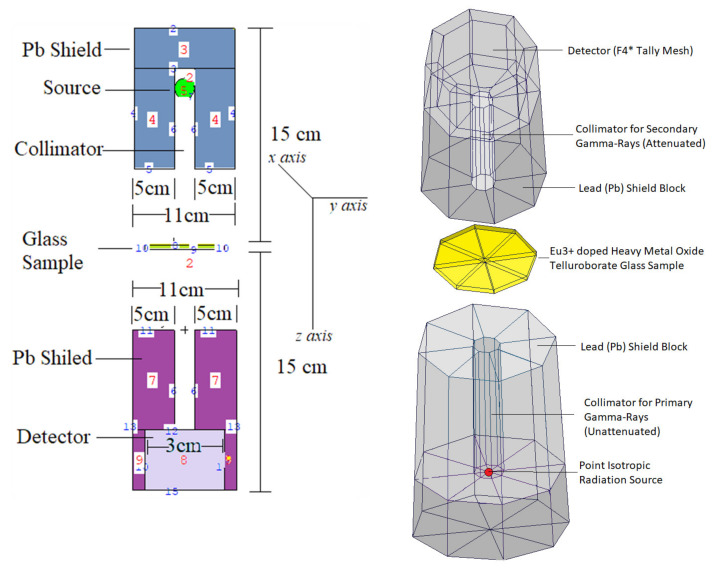
MCNPX simulation setup used for gamma-ray transmission simulations (A direct screenshot from the MCNPX Visual Editor VE X_22S).

The review of the literature showed that researchers are very interested in the use of mathematical simulation techniques in material sciences and radiation studies [23,24,25]. Among the well-known Monte Carlo codes, MCNPX is a general-purpose Monte Carlo software that enables the modeling of many radiation transport categories in nuclear, medical, and particle physics research. This software allows the user to provide the required simulation parameters, such as geometrical features and material properties, and the energy and type of radiation source, such as narrow beam, point isotropic, and so forth. In this study, we aimed to generate a useful gamma-ray transmission setup to assess the transmission parameters of AL glasses. Most importantly, we aimed to evaluate each glass sample with a different composition to see the variation in gamma-ray attenuation properties depending on the composition of the glass and the substitution percentage. Accordingly, we developed a generic gamma-ray transmission configuration in our research that may give beneficial information such as the intensity of the attenuated gamma ray when calculating the linear attenuation coefficients. Our study constructed a basic gamma-ray transmission setup that can provide essential information about the attenuated gamma-intensity rays when computing the linear attenuation coefficients. As a result, an INPUT file including cell card, surface card, and source information was generated. We identified the cells that were used and their densities and boundaries on the cell card. The *x*, *y*, and *z*-axis locations were used to specify the geometric characteristics of the boundaries in the surface card. The modeled simulation setup is shown in Figure 1, together with its three-dimensional and two-dimensional perspectives derived using the MCNPX visual editor. As shown in Figure 1, we located the equipment based on its function in a gamma-ray transmission simulation. For example, a gamma-ray attenuator was positioned between the source of isotropic point radiation and the detecting field (F4 Tally Mesh). Additionally, two major lead (Pb) blocks were constructed to absorb scattering gamma rays, perhaps improving detection consistency. We want to highlight that those simulations on each glass sample were conducted using photon energies ranging from 0.015 MeV to 15 MeV. Finally, each glass sample was exposed to a total of 10^8^ particle tracks (Number of History) with varying photon intensities. After completing all simulations, the MCNPX output had a less than 1% relative error rate. MCNPX simulations were run on a Lenovo^TM^ ThinkStation620 equipped with a Ryzen^TM^ Threadripper^TM^ Pro 3995WX CPU (2.7 GHz, 64 Cores, 256 MB Cache). Following that, the attenuation parameters were determined using the Phy-X/PSD platform. The main differentiation between the MCNPX and the Phy-X/PSD formats is their architecture.

On the one hand, MCNPX is a Monte Carlo technique that requires user-defined parameters for every component of the system, including material design, mathematical model, physics list utilized, and variance reduction methods. Phy-X/PSD, on the other hand, is a platform that instantaneously returns results as a result of mathematical calculations conducted against a preset database. Additional support is presented in the original paper, which is also available online [22].

### 2.2. Investigated Nuclear Shielding Parameters

Following the determination of the linear attenuation coefficients (µ), many additional gamma radiation attenuation parameters were determined. To begin, we calculated the mass attenuation coefficients (µ) of the AL glasses using Equation (1) [26],

μ_m_ = μ/ρ (1)

where µ is the linear attenuation coefficient and ρ is the glass density. Following that, the half-value layer (T1/2) values for the SM glasses were computed. T_1/2_ is a critical element in evaluating the need to use very severe radiation protection measures [27]. This value indicates the thickness of a shielding material at which the intensity of a gamma-ray incident on it is effectively halved. Additionally, critical gamma-ray shielding properties such as mean free path (λ) [28] and effective atomic numbers (Z_eff_) [29] were evaluated in relation to gamma-ray attenuation, exposure, and energy absorption build-up factors (EBF and EABF) [30,31,32,33]. On the other hand, we sought to assess the attenuation characteristics of SM glasses against fast neutrons, which may be advantageous for specific applications in neutron-based (i.e., photodisintegration) radiation facilities. As a consequence, we computed the effective cross-sections for the removal of fast neutrons in AL glasses. The literature and other sources provide detailed information on the factors under study [34,35,36].

## 3. Results and Discussion

Five distinct Eu^3+^ reinforced heavy metal oxide glasses with a nominal composition of 55B_2_O_3_ + 19.5TeO_2_ + 10K_2_O + (15x) PbO + xAl_2_O_3_ + 0.5Eu_2_O_3_ were studied in terms of their gamma-ray and neutron shielding performances. To begin, we derived the linear attenuation coefficients using the MCNPX algorithm. Then, following the procedure outlined above, linear attenuation coefficients for each glass sample were obtained at various energies ranging from 0.015 MeV to 15 MeV. The identical glass mixtures (see Table 1) were then designed in the Phy-X/PSD platform together with corresponding densities. In general, the acquired findings verified one another.

**Figure 2:**
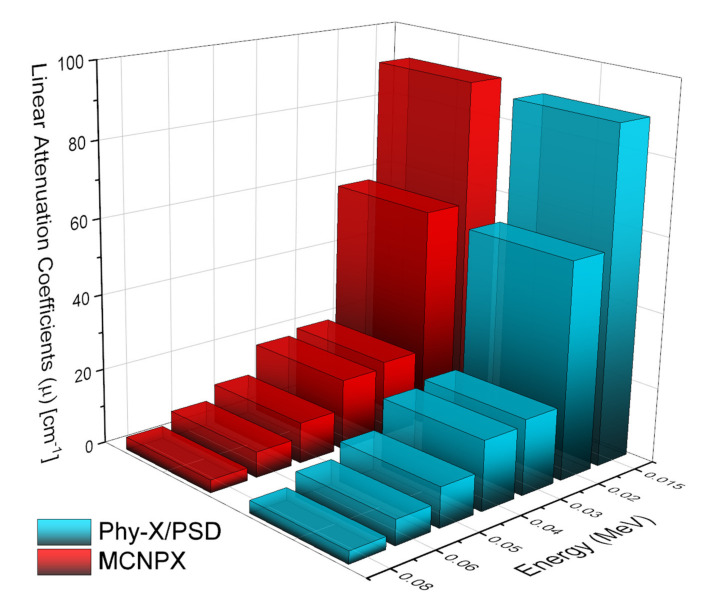
Comparison of linear attenuation coefficient (µ) values for AL0.0 sample obtained from MCNPX and Phy-X/PSD at low gamma-ray energy region.

Figure 2 includes the comparison-provided linear attenuation coefficients from MCNPX and Phy-X/PSD for AL0.0 sample in the low energy area. As shown in Figure 2, a significant connection exists between the two findings. We found, however, a few small discrepancies between the obtained results. Given the diametrical opposition between the MCNPX and Phy-X/PSD formats, it is quite fair to expect some differences in the findings.

**Figure 3:**
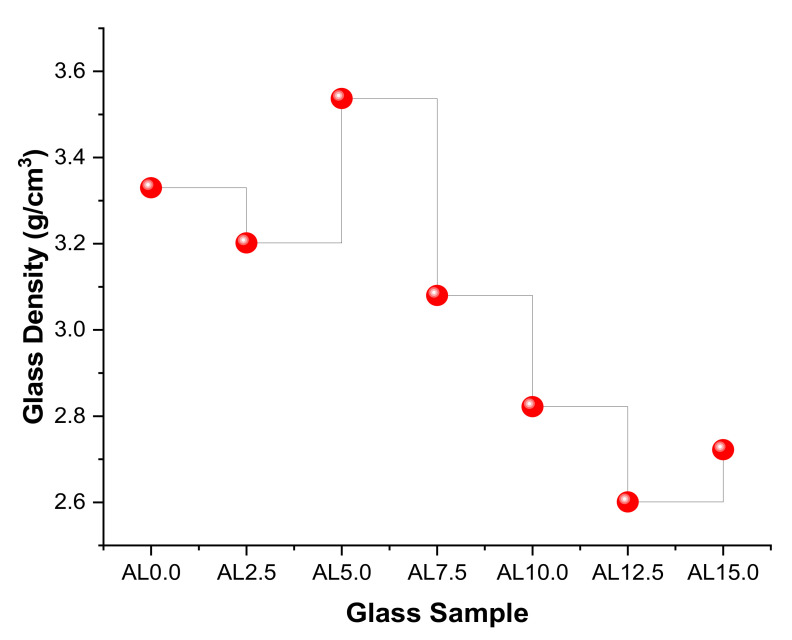
Variation of glass densities as a function of glass type (i.e., Al_2_O_3_ %mole).

**Figure 4:**
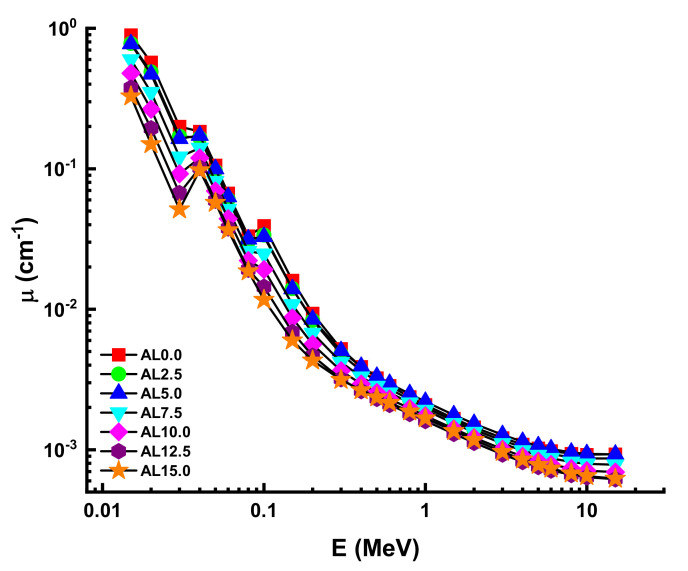
Variation of linear attenuation coefficient (µ) against photon energy for all glasses.

Figure 3 depicts the glass densities of the studied samples, encoded AL0.0, AL2.5, AL5.0, AL7.5, AL10, AL12.5, and AL15.0, respectively. It is observed that reducing the quantity of PbO in the glass structure also resulted in a noticeable decrease in density. Due to the fact that the density term has a direct connection with the linear attenuation coefficients (µ) of shields, we sought to understand the variations in µ values as a function of density variation. Accordingly, we demonstrated the variation of µ values as a function of photon energy in the 0.015 MeV to 15 MeV range. A rapid decrement was observed in the low-energy region, where the photoelectric effect is the main interaction process between the ionizing gamma rays and the material. We would like to highlight that the glass samples’ findings were sharply different due to the high molar differences in the PbO/Al_2_O_3_ substitution. Nonetheless, adding the Al_2_O_3_ additive decreased the linear attenuation coefficients of AL glasses in a synergistic manner. Our results demonstrated that the AL0.0 sample had the highest values at all energies due to the highest PbO contribution in the structure. One can say that a decreasing PbO amount in Eu^3+^ based HMO glasses might decrease the linear attenuation properties even though it would increase the direct or indirect band gap [20] and the luminescence properties of the HMO glasses, as seen in Figure 4. In this instance, linear attenuation coefficients were reported as 89.326 cm^−1^, 77.997 cm^−1^, 77.436 cm^−1^, 59.836 cm^−1^, 47.865 cm^−1^, 37.703 cm^−1^, and 32.745 cm^−1^ for AL.00, AL2.5, AL5.0, AL7.5, AL10, AL12.5, and AL15.0 at 0.015 MeV, respectively.

**Figure 5:**
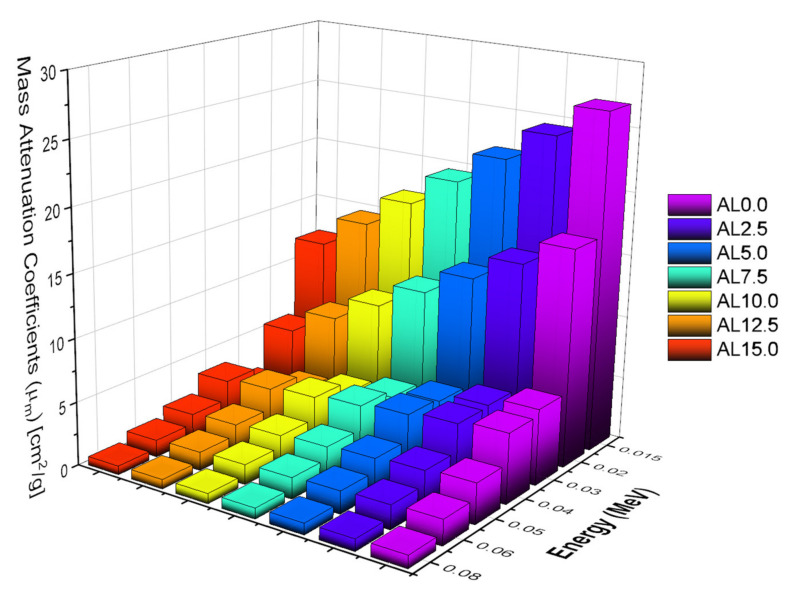
Variation of mass attenuation coefficients (µ_m_) at low-energy region.

**Figure 6:**
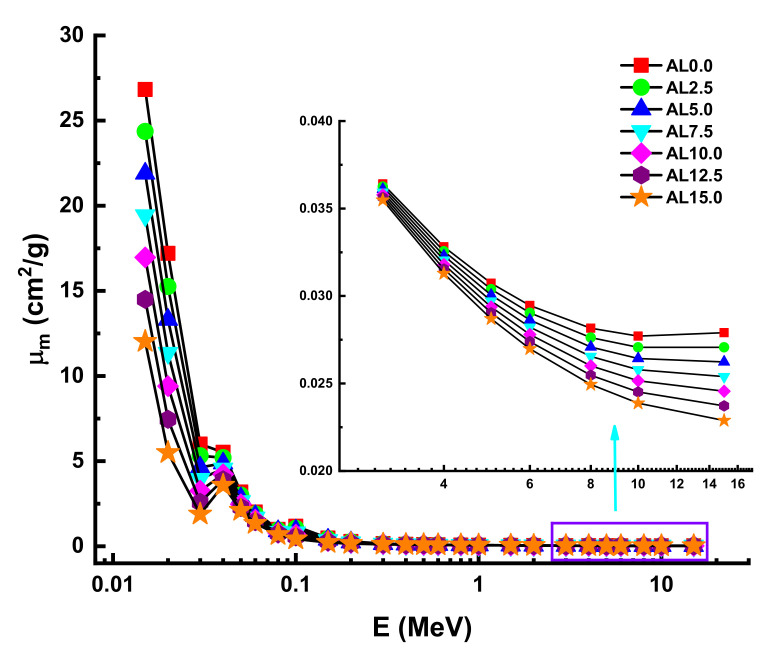
Variation of mass attenuation coefficient (µ_m_) against photon energy for all glasses.

The term mass attenuation coefficient (µ_m_), on the other hand, refers to the density-independent attenuation coefficient, which includes important information about the material’s shielding effectiveness as a function of its elemental composition. The fluctuation of µ_m_ values in the low-energy area is shown in Figure 5. As was the case with the finding of µ values, the AL0.0 sample was reported with maximum µ_m_ values. This is because the substituted PbO and Al_2_O_3_ have distinct elemental structures and atomic numbers, which directly impact their gamma-ray attenuation characteristics. A linear change line was found in the low-energy area. As shown in Figure 6, the AL0.0 sample was presented with the highest µ_m_ values. Additionally, we showed the change of µ_m_ values from 0.015 MeV to 15 MeV as a function of photon energy. In addition to dominated energy regions, such as low, mid, and high energy, a visible difference was also reported in terms of µ_m_ differences of the AL glasses. Our findings clearly indicated that AL0.0 has the maximum µ_m_ values at all energy regions. 

**Figure 7:**
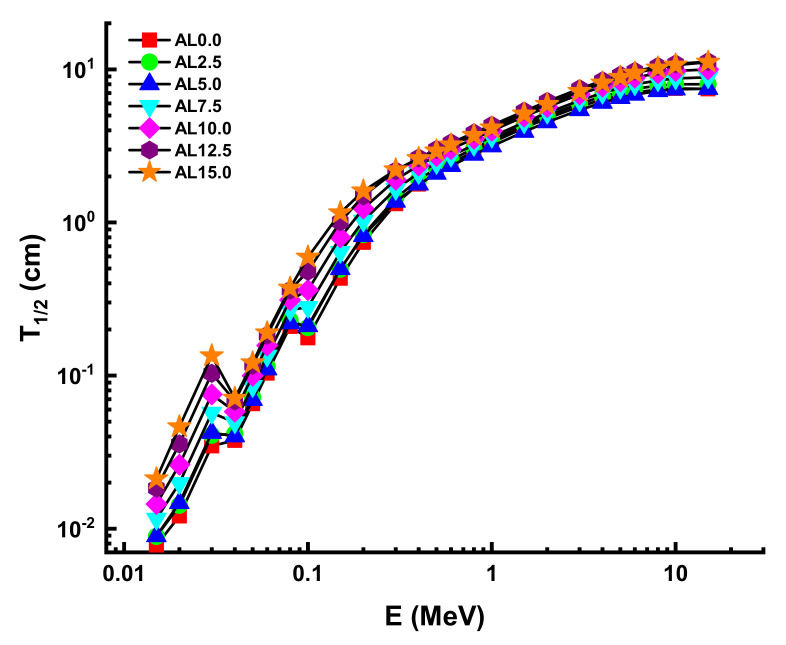
Variation of half-value layer (T_1/2_) against photon energy for all glasses.

The term half-value thickness (T_1/2_) refers to the thickness of a shielding material that is capable of quantitatively halving the intensity of interacted gamma rays on it. As a result, knowledge of T_1/2_ and the energy values examined in the radiation field enables the most accurate radiation protection and safety measures to be provided. To provide the gamma-ray attenuation properties of AL glasses from that point, we determined the T_1/2_ in a wide range of gamma ray energy. However, it is worth noting that T_1/2_ value has an inverse relationship with µ value. Thus, materials with higher µ values would have lower T_1/2_ values, suggesting better gamma-ray attenuation. Figure 7 demonstrates the variation of T_1/2_ values against photon energy for all AL glasses. Therefore, we would like to define some quantitative T_1/2_ values in terms of the physical usage of this kind of glass, as well as a better understanding of the alterations caused by the PbO/AL_2_O_3_ replacement. For example, T_1/2_ values were reported as 0.17653 cm, 0.20536 cm, 0.21091 cm, 0.27985 cm, 0.36164 cm, 0.48084 cm, and 0.59325 cm for AL0.0, AL2.5, AL5.0, AL7.5, AL10, AL12.5, and AL15.0 at 0.1 MeV, respectively. To serve as a practical example, one can say that a 15% mole PbO/AL_2_O_3_ substitution can increase the T_1/2_ values to 0.41672 cm (almost half a centimeter) at 0.1 MeV. 

**Figure 8:**
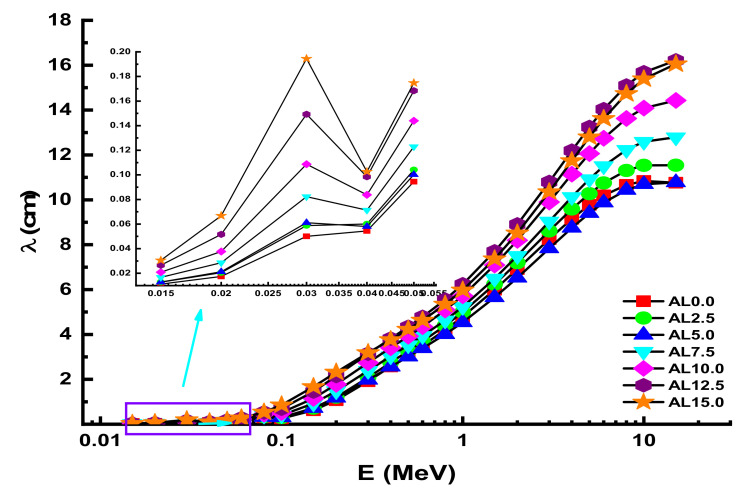
Variation of mean free path (λ) against photon energy for all glasses.

**Figure 9:**
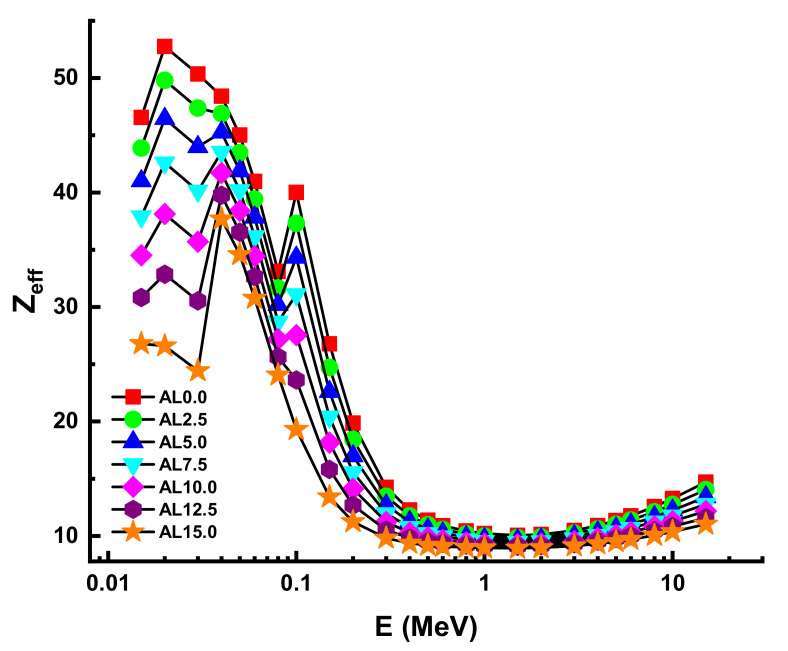
Variation of effective atomic number (Z_eff_) against photon energy for all glasses.

Particles collide when they travel through a substance, altering their direction of motion. Thus, the average distance between these encounters provides information on the likelihood of a specific interaction. This distance is often referred to as the mean free path (λ). This fundamental parameter was also determined for AL glasses at 0.015–15 MeV photon energy. The behavioral view of λ values as a function of incident photon energy can be observed in Figure 8. Due to the material’s high density, the AL0.0 sample has the smallest average distance (λ) between the two collisions, which is another indication of excellent shielding capabilities. On the other hand, Z_eff_ is shown in Figure 9 as a function of incoming photon energy at all energies. According to the findings, the sample with the greatest Z_eff_ value is AL0.0, which has the greatest PbO contribution in its structure. However, some significant differences were seen owing to the samples’ major structural differences. This can be explained by the total elemental structure of the substituted PbO and Al_2_O_3_. The buildup factor is a reference method used throughout shielding computations to accommodate for scattered radiation and any secondary particles present in the environment. To compensate for secondary radiation accumulation, a buildup factor must be included. Thus, the accumulation factor is a multiplicative factor that includes the contribution of scattered photons to the un-collided photons’ response. 

**Figure 10:**
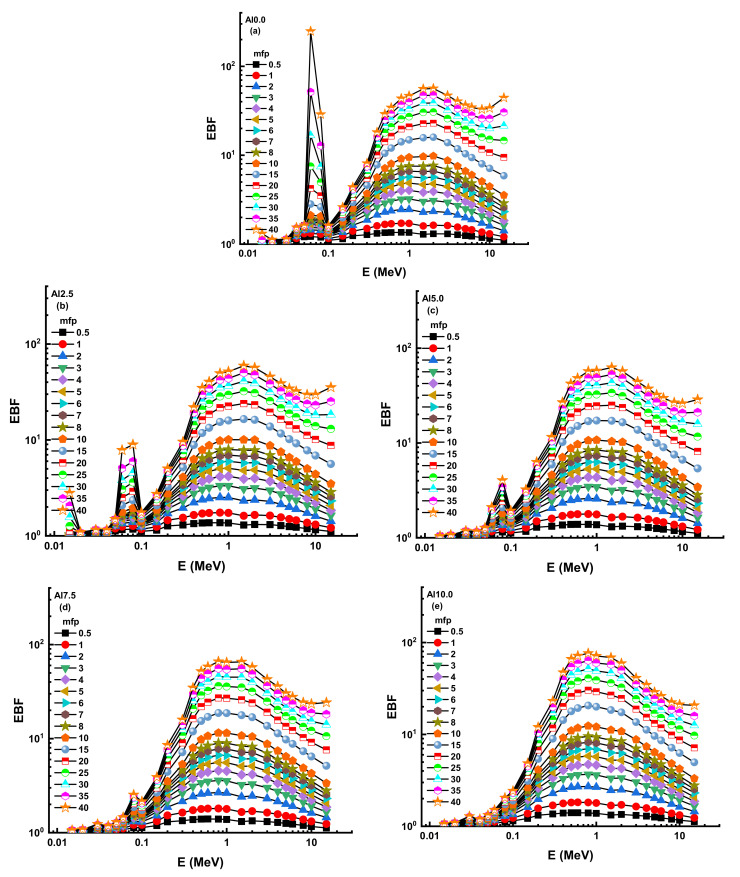

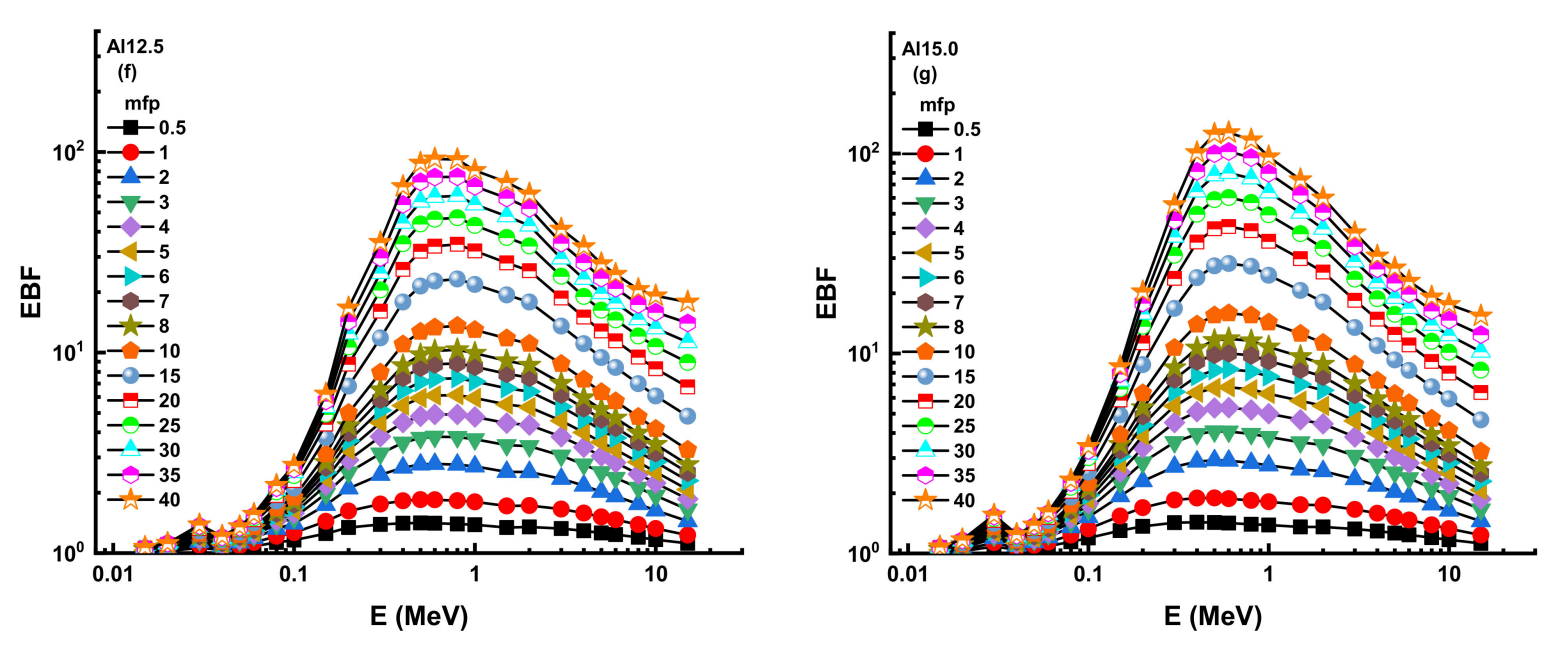
Variation of exposure buildup factor (EBF) against photon energy for all glasses. (**a**) AL0.0; (**b**)AL2.5; (**c**)AL5.0; (**d**) AL7.5; (**e**) AL10.0; (**f**) AL12.5; (**g**) AL15.0.

**Figure 11:**
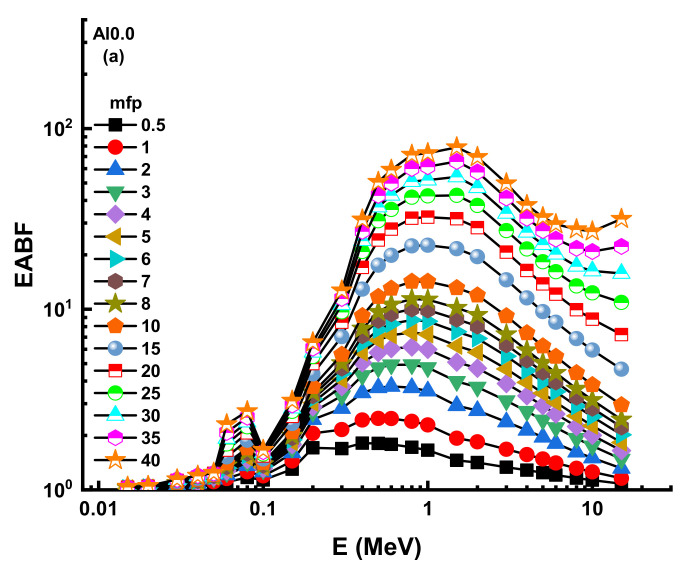

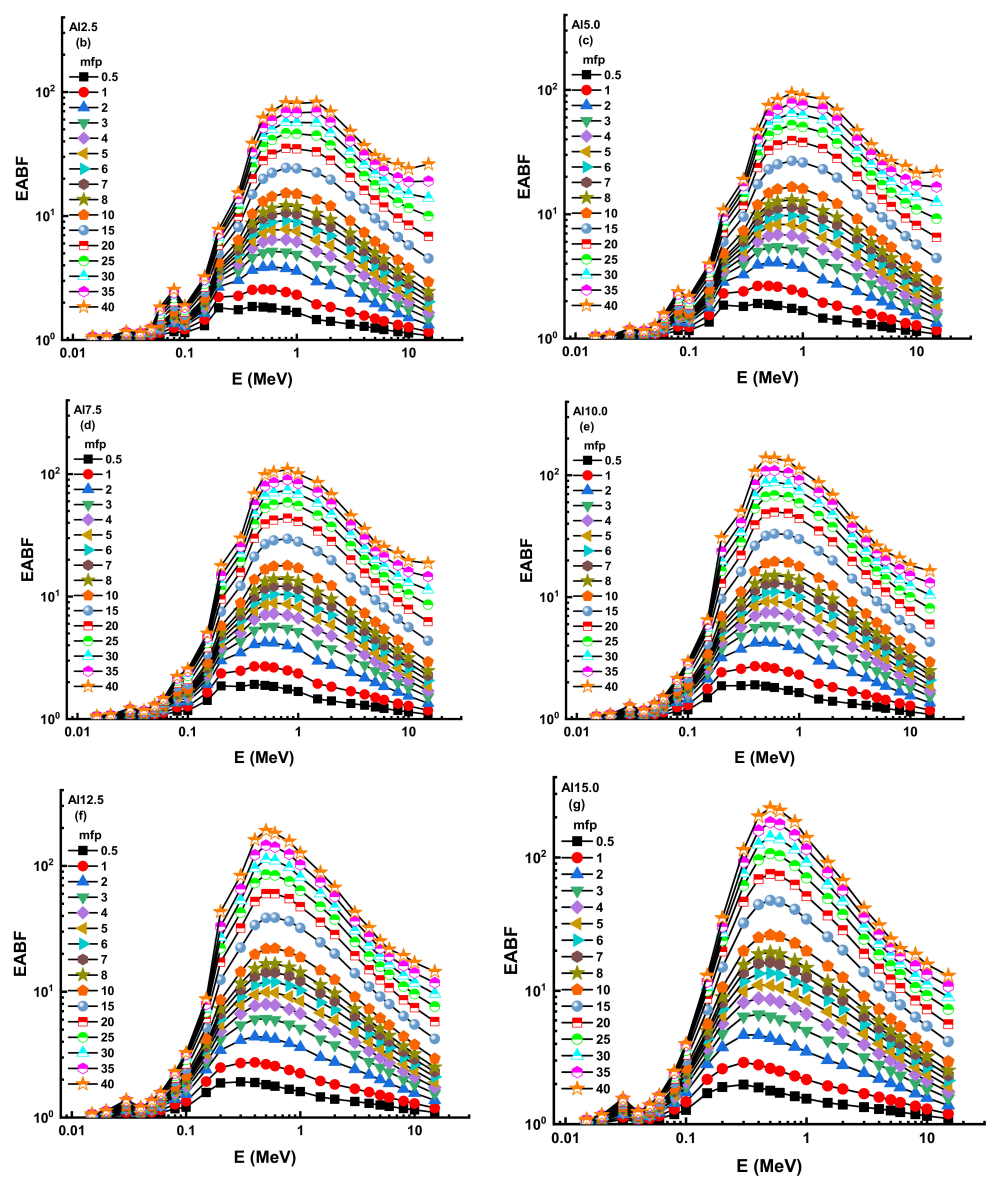
Variation of energy absorption buildup factor (EABF) against photon energy for all glasses. (**a**) AL0.0; (**b**)AL2.5; (**c**)AL5.0; (**d**) AL7.5; (**e**) AL10.0; (**f**) AL12.5; (**g**) AL15.0.

Consequently, one may compute the accumulation factor as the ratio of the total exposure to the un-collided dose-response [37]. Additionally, there are two main types of buildup factors that can be listed as the exposure buildup factor (EBF) and the energy absorption buildup factor (EABF) [38,39]. In the framework of the earlier explanation, the smaller buildup factor values may be interpreted as a pattern of dominance against gamma rays since the quantity of un-collided photons in successful shields is small. Figure 10 and Figure 11 illustrate the progression of the EBF and EABF values as a function of energy along different mean free routes (i.e., from 0.5 to 40).

The greatest ratios were seen in both EBF and EABF at intermediate energies when Compton scattering was the primary interaction between the gamma ray and the material. In other words, this region has a large number of photons that have not collided. As a consequence, significantly larger accumulation factor values are needed to adjust the transmission computations. Our findings show that AL0.0 has the lowest EBF and EABF accumulation factors (see Supplementary Tables S1–S7). As a result, incoming gamma rays may interact more often with the AL0.0 sample than with other samples.

**Figure 12:**
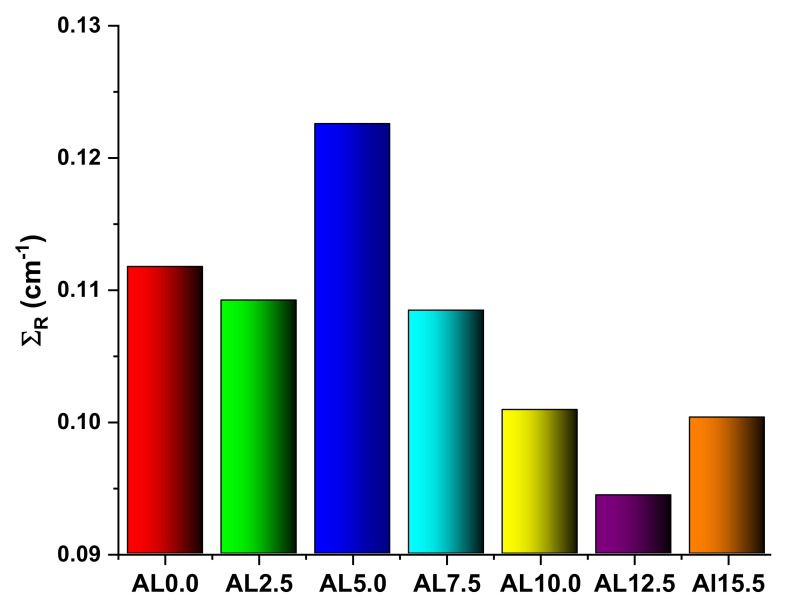
Effective removal cross-sections for fast neutrons (Σ_R_) for all glasses.

The effective removal cross-section, (Σ_R_, cm^2^/g), represents the probability that a fast or fission energy neutron might encounter a first collision, thus removing it from the category of penetrating, un-collided neutrons. It is expected to remain nearly constant for neutron energy between 2 and 12 MeV. Additionally, substances with the highest Σ_R_ values may be regarded better in terms of resistance to dangerous neutron particles. The Σ_R_ values of AL glasses were evaluated in this investigation, and the outcomes are shown in Figure 12. Following the gamma-ray attenuation characteristics, it was discovered that adding Al_2_O_3_ has a distinct synergistic effect. Therefore, one might argue that Al_2_O_3_ is an unpractical tool for enhancing the gamma-ray and fast neutron shielding characteristics of HMO glasses, as shown in Figure 12. 

**Figure 13:**
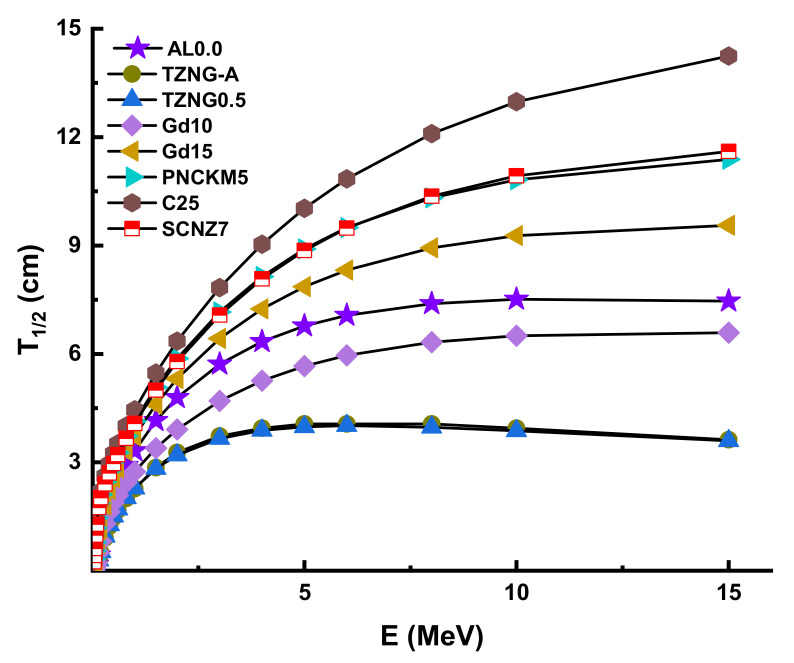
Half-value layer comparison between some glasses and AL0.0 sample.

**Figure 14:**
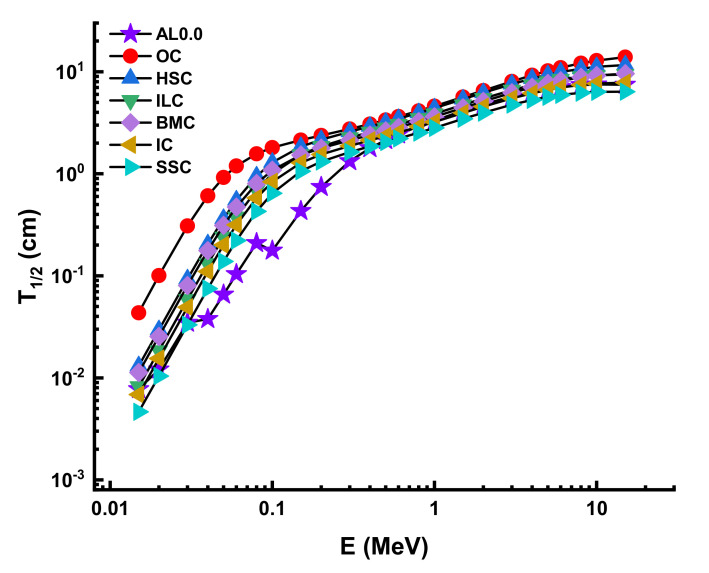
Half-value layer comparison between some concretes and AL0.0 sample.

Finally, we evaluated the shielding characteristics of Al0.0 with those of other shielding materials, including TZNG-A [40], TZNG0.5 [27], Gd10 [41], Gd15 [42], PNCKM5 [43], C25 [44], SCNZ7 [45], Ordinary Concrete (OC), HSC, ILC, BMC, IC, and SSC [46,47,48]. The objective of this research was to get a better understanding of the AL0.0 sample’s comprehensive results in terms of the T_1/2_ values required to suppress incoming gamma-ray photons from 0.015 to 15 MeV. Consequently, it turns out that the T1/2 values of the AL0.0 sample are less than Gd15, PNCKM5, C25, and SCNZ7, and that it therefore has shielding properties higher than TZNG-A, TZNG0.5, and Gd10 samples with higher shielding properties. Following this, the T_1/2_ values of the AL0.0 sample were determined to be those of various types of standard/special concrete. Figure 13 illustrates the variance in T_1/2_ values for Al0.0 and various glass shields as a function of incoming photon energy. AL0.0 has the lowest T_1/2_ values when compared to TZNG-A, TZNG0.5, Gd10, Gd15, PNCKM5, C25, and SCNZ7 glasses at low energy range, which have all been investigated before as potential glass shields. Finally, we discussed our results regarding the T_1/2_ values for AL0.0 and other kinds of concrete. Figure 14 illustrates the results as a function of incoming photon energy. The AL0.0 sample was found to have the lowest T_1/2_ values in general. Thus, an AL0.0 glass sample may be an appropriate option in certain radiation facilities where conventional and new-generation concretes are utilized for operational shielding applications.

## 4. Conclusions

The shielding characteristics of heavy metal oxide telluborate glasses containing Eu_2_O_3_ were examined in this research. According to a study of the literature, borate- and tellurium-based glasses are the most researched. However, heavy metal oxide doped glasses have a broad range of uses due to their high transmittance in the visible and mid-infrared regions, high refractive indices, and excellent thermal, electrical, and chemical endurance. Additionally, the high densities of heavy metal oxide glasses contribute to their radiation shielding properties. Indeed, doping these glasses with denser lanthanide oxides results in substantial increases in the densities of the produced glasses. According to the research on which our analysis is based, the densities of the samples varied between 2.601 g/cm^3^ and 3.537 g/cm^3^. The sample containing 5% Al_2_O_3_ by weight exhibited the greatest density value. Therefore, the highest value of this sample’s effective removal cross-sections may be interpreted as a change in the density value. However, when we examine that the AL0.0 sample with the greatest PbO concentration had the highest effective values for all gamma-ray shielding parameters, it can be concluded that these values are not only density-dependent but also directly linked to the nature of these compounds. It can be also concluded that using PbO in the Eu^3+^ doped heavy metal oxide glasses could be a useful tool to keep gamma-ray shielding properties at a maximum level. 

## Figures and Tables

**Table 1:** Elemental compositions (%mole) of the studied glass samples.

Code	B_2_O_3_	TeO_2_	K_2_O	PbO	Al_2_O_3_	Eu_2_O_3_	ρ (g/cm^3^)
**AL0.0**	0.550	0.195	0.100	0.150	0.000	0.005	3.33
**AL2.5**	0.550	0.195	0.100	0.125	0.025	0.005	3.202
**AL5.0**	0.550	0.195	0.100	0.100	0.050	0.005	3.537
**AL7.5**	0.550	0.195	0.100	0.075	0.075	0.005	3.08
**AL10.0**	0.550	0.195	0.100	0.050	0.100	0.005	2.822
**AL12.5**	0.550	0.195	0.100	0.025	0.125	0.005	2.601
**AL15.0**	0.550	0.195	0.100	0.000	0.150	0.005	2.722

## Data Availability

The data presented in this study are available on request from the corresponding author.

## Supplementary Materials

The following are available online at https://www.mdpi.com/article/10.3390/ma14185334/s1, Table S1. (EBF and EABF) G–P fitting coefficients (b, c, a, Xk and d) of Al0.0 glass sample, Table S2. (EBF and EABF) G–P fitting coefficients (b, c, a, Xk and d) of AL2.5 glass sample, Table S3. (EBF and EABF) G–P fitting coefficients (b, c, a, Xk and d) of AL5.0 glass sample, Table S4. (EBF and EABF) G–P fitting coefficients (b, c, a, Xk and d) of AL7.5 glass sample, Table S5. (EBF and EABF) G–P fitting coefficients (b, c, a, Xk and d) of AL10.0 glass sample, Table S6. (EBF and EABF) G–P fitting coefficients (b, c, a, Xk and d) of AL12.5 glass sample, Table S7 (EBF and EABF) G–P fitting coefficients (b, c, a, Xk and d) of AL15.0 glass sample.
