# Supplementary material for: A Closer Look on Nuclear Radiation Shielding Properties of Eu3+ Doped Heavy Metal Oxide Glasses: Impact of Al2O3/PbO Substitution
Source: Materials (Basel). 2021 Sep 16;14(18):5334. doi: 10.3390/ma14185334 (PMC8466938; doi:10.3390/ma14185334)
Supplement: Supplementary file 1 [file materials-14-05334-s001.zip › materials-1355948-supplementary.pdf]

Table S1. (EBF and EABF) G–P fitting coefficients (b, c, a, Xk and d) of Al0.0 glass sample.

Energy (MeV)	Z_{eq}	G-P Fitting Parameters for EBF					G-P Fitting Parameters for EABF				
		a	b	c	d	X_k	a	b	c	d	X_k
0.015	19.30	0.435	1.010	0.274	-0.387	10.046	0.302	1.009	0.335	-0.267	11.982
0.020	22.00	0.340	1.015	0.328	-0.277	11.071	0.273	1.014	0.329	-0.220	13.786
0.030	22.89	0.207	1.042	0.373	-0.220	20.684	0.245	1.041	0.340	-0.169	15.927
0.040	29.44	0.233	1.160	0.330	-0.094	14.814	0.193	1.056	0.389	-0.279	25.359
0.050	30.31	0.176	1.314	0.315	-0.117	12.779	0.196	1.108	0.317	-0.109	12.343
0.060	30.82	0.379	1.322	0.305	-0.137	14.193	0.340	1.155	0.288	-0.157	14.592
0.080	31.57	0.324	1.297	0.351	-0.143	14.068	0.298	1.258	0.323	-0.147	14.310
0.100	41.94	0.289	1.159	0.301	-0.155	13.751	0.327	1.195	0.254	-0.168	13.813
0.150	43.60	0.196	1.236	0.455	-0.104	14.319	0.317	1.445	0.291	-0.176	14.049
0.200	44.57	0.172	1.402	0.509	-0.097	14.402	0.321	2.057	0.299	-0.196	13.993
0.300	45.75	0.093	1.499	0.691	-0.046	14.363	0.182	2.156	0.508	-0.107	13.942
0.400	46.46	0.054	1.607	0.831	-0.039	14.157	0.133	2.436	0.642	-0.099	13.887
0.500	46.92	0.033	1.670	0.910	-0.031	14.159	0.092	2.485	0.751	-0.077	13.881
0.600	47.23	0.017	1.694	0.964	-0.023	13.994	0.071	2.480	0.813	-0.065	13.740
0.800	47.56	0.003	1.718	1.020	-0.017	14.070	0.045	2.393	0.894	-0.050	13.639
1.000	47.68	-0.003	1.712	1.046	-0.015	13.430	0.031	2.289	0.938	-0.042	13.514
1.500	44.90	-0.027	1.603	1.146	-0.001	9.873	-0.005	1.932	1.071	-0.018	13.543
2.000	37.01	-0.021	1.633	1.125	-0.005	11.349	-0.005	1.845	1.070	-0.018	11.847
3.000	28.16	-0.004	1.620	1.059	-0.014	12.151	0.001	1.676	1.039	-0.018	12.318
4.000	25.40	0.005	1.555	1.024	-0.019	12.851	0.014	1.567	0.994	-0.029	13.791
5.000	24.17	0.013	1.490	1.002	-0.025	13.145	0.023	1.482	0.967	-0.037	14.262
6.000	23.44	0.023	1.449	0.973	-0.033	13.299	0.025	1.408	0.964	-0.037	13.802
8.000	22.69	0.030	1.365	0.958	-0.039	13.583	0.034	1.315	0.945	-0.040	13.159
10.000	22.36	0.039	1.306	0.940	-0.048	13.757	0.043	1.257	0.926	-0.050	14.063
15.000	22.09	0.050	1.211	0.932	-0.057	14.084	0.039	1.160	0.956	-0.044	14.531

Table S2. (EBF and EABF) G–P fitting coefficients (b, c, a, Xk and d) of AL2.5 glass sample.

Energy (MeV)	Z_{eq}	G-P Fitting Parameters for EBF					G-P Fitting Parameters for EABF				
		a	b	c	d	X_k	a	b	c	d	X_k
0.015	18.62	0.439	1.011	0.268	-0.491	18.170	0.316	1.010	0.326	-0.379	19.167
0.020	21.07	0.267	1.015	0.379	-0.190	10.989	0.261	1.016	0.348	-0.201	12.642
0.030	21.93	0.213	1.047	0.373	-0.188	17.764	0.242	1.046	0.347	-0.162	14.930
0.040	28.78	0.240	1.043	0.330	-0.098	14.256	0.201	1.039	0.388	-0.278	24.651
0.050	29.66	0.209	1.194	0.334	-0.129	12.834	0.219	1.090	0.335	-0.125	12.612
0.060	30.16	0.329	1.246	0.326	-0.136	13.961	0.308	1.143	0.306	-0.154	14.570
0.080	30.91	0.291	1.270	0.375	-0.135	14.044	0.278	1.255	0.341	-0.141	14.330
0.100	40.17	0.275	1.174	0.325	-0.148	13.767	0.304	1.217	0.287	-0.159	14.469
0.150	41.76	0.173	1.245	0.493	-0.090	14.413	0.286	1.457	0.323	-0.159	14.091
0.200	42.71	0.173	1.454	0.513	-0.099	14.322	0.319	2.217	0.305	-0.198	14.019
0.300	43.86	0.086	1.543	0.712	-0.045	14.425	0.170	2.270	0.533	-0.102	14.000
0.400	44.55	0.047	1.650	0.856	-0.036	14.166	0.122	2.537	0.672	-0.094	13.896
0.500	45.01	0.027	1.708	0.936	-0.028	14.223	0.083	2.564	0.781	-0.073	13.888
0.600	45.29	0.013	1.731	0.985	-0.021	13.975	0.062	2.541	0.841	-0.061	13.747
0.800	45.62	0.000	1.747	1.038	-0.015	14.053	0.038	2.430	0.918	-0.047	13.646
1.000	45.75	-0.006	1.735	1.061	-0.013	13.430	0.026	2.315	0.958	-0.039	13.525
1.500	42.74	-0.029	1.619	1.157	0.000	7.849	-0.009	1.939	1.087	-0.016	13.489
2.000	34.56	-0.021	1.650	1.124	-0.005	10.663	-0.009	1.842	1.082	-0.014	11.186
3.000	26.18	-0.005	1.626	1.059	-0.013	12.004	0.002	1.683	1.035	-0.018	12.438
4.000	23.71	0.006	1.560	1.020	-0.018	12.620	0.015	1.573	0.990	-0.028	13.323
5.000	22.63	0.014	1.496	0.995	-0.024	13.168	0.022	1.488	0.966	-0.035	14.373
6.000	21.98	0.023	1.453	0.968	-0.032	13.255	0.027	1.416	0.956	-0.037	13.472
8.000	21.32	0.031	1.371	0.951	-0.038	13.552	0.035	1.322	0.938	-0.038	12.774
10.000	21.01	0.038	1.310	0.937	-0.045	13.670	0.045	1.267	0.913	-0.051	13.978
15.000	20.79	0.050	1.216	0.922	-0.056	13.978	0.036	1.164	0.957	-0.040	14.494

Table S3. (EBF and EABF) G–P fitting coefficients (b, c, a, Xk and d) of AL5.0 glass sample.

Energy (MeV)	Z_{eq}	G-P Fitting Parameters for EBF					G-P Fitting Parameters for EABF				
		a	b	c	d	X_k	a	b	c	d	X_k
0.015	17.90	0.150	1.009	0.485	-0.284	28.480	0.149	1.009	0.485	-0.279	28.524
0.020	20.07	0.186	1.016	0.437	-0.092	10.897	0.248	1.017	0.368	-0.179	11.355
0.030	20.89	0.220	1.052	0.372	-0.151	14.483	0.239	1.051	0.356	-0.153	13.811
0.040	28.10	0.242	1.047	0.332	-0.103	13.645	0.212	1.044	0.374	-0.243	22.139
0.050	28.99	0.243	1.071	0.353	-0.141	12.895	0.243	1.071	0.353	-0.141	12.896
0.060	29.49	0.277	1.168	0.348	-0.135	13.722	0.275	1.130	0.326	-0.152	14.547
0.080	30.23	0.257	1.242	0.400	-0.126	14.018	0.257	1.252	0.360	-0.134	14.351
0.100	38.25	0.258	1.191	0.352	-0.139	13.785	0.279	1.241	0.325	-0.148	15.213
0.150	39.77	0.163	1.282	0.517	-0.085	14.357	0.275	1.546	0.343	-0.156	14.013
0.200	40.68	0.163	1.493	0.542	-0.094	14.232	0.301	2.308	0.338	-0.189	13.980
0.300	41.78	0.079	1.593	0.737	-0.042	14.462	0.157	2.394	0.564	-0.097	14.042
0.400	42.45	0.040	1.700	0.885	-0.034	14.178	0.109	2.653	0.706	-0.088	13.908
0.500	42.88	0.019	1.754	0.965	-0.025	14.298	0.072	2.656	0.815	-0.067	13.896
0.600	43.17	0.007	1.772	1.010	-0.019	13.953	0.053	2.612	0.874	-0.056	13.755
0.800	43.49	-0.005	1.780	1.058	-0.014	14.034	0.031	2.473	0.946	-0.043	13.654
1.000	43.60	-0.010	1.763	1.079	-0.012	13.430	0.019	2.345	0.982	-0.035	13.539
1.500	40.35	-0.031	1.636	1.164	0.002	8.342	-0.013	1.942	1.099	-0.014	12.899
2.000	31.94	-0.021	1.670	1.123	-0.005	9.871	-0.014	1.839	1.096	-0.010	10.424
3.000	24.19	-0.007	1.631	1.062	-0.011	12.333	0.000	1.685	1.039	-0.016	12.313
4.000	22.03	0.006	1.566	1.015	-0.018	12.375	0.016	1.580	0.985	-0.027	12.827
5.000	21.09	0.014	1.503	0.988	-0.024	13.192	0.022	1.494	0.964	-0.033	14.491
6.000	20.54	0.023	1.457	0.964	-0.031	13.208	0.028	1.425	0.947	-0.036	13.127
8.000	19.96	0.031	1.376	0.943	-0.036	13.518	0.035	1.330	0.932	-0.036	12.370
10.000	19.70	0.037	1.314	0.932	-0.042	13.580	0.048	1.277	0.899	-0.052	13.904
15.000	19.51	0.049	1.219	0.918	-0.054	13.704	0.037	1.171	0.946	-0.040	14.432

Table S4. (EBF and EABF) G–P fitting coefficients (b, c, a, Xk and d) of AL7.5 glass sample.

Energy (MeV)	Z_{eq}	G-P Fitting Parameters for EBF					G-P Fitting Parameters for EABF				
		a	b	c	d	X_k	a	b	c	d	X_k
0.015	17.16	0.215	1.012	0.414	-0.272	21.741	0.206	1.012	0.414	-0.236	22.112
0.020	18.99	0.289	1.024	0.289	-0.146	12.435	0.363	1.023	0.250	-0.254	12.544
0.030	19.77	0.223	1.059	0.375	-0.117	11.731	0.239	1.059	0.360	-0.144	12.752
0.040	27.41	0.244	1.050	0.333	-0.108	13.004	0.223	1.048	0.360	-0.207	19.500
0.050	28.30	0.241	1.077	0.356	-0.140	13.142	0.243	1.079	0.351	-0.140	13.229
0.060	28.81	0.235	1.111	0.367	-0.134	13.579	0.249	1.124	0.342	-0.149	14.540
0.080	29.54	0.221	1.212	0.426	-0.117	13.991	0.235	1.249	0.379	-0.128	14.373
0.100	36.15	0.239	1.210	0.384	-0.130	13.807	0.249	1.270	0.369	-0.136	16.071
0.150	37.57	0.152	1.324	0.546	-0.080	14.291	0.263	1.650	0.366	-0.153	13.922
0.200	38.42	0.144	1.527	0.591	-0.084	14.129	0.270	2.358	0.395	-0.173	13.891
0.300	39.46	0.067	1.635	0.782	-0.039	14.157	0.139	2.477	0.620	-0.089	13.848
0.400	40.09	0.031	1.736	0.922	-0.031	13.838	0.093	2.693	0.759	-0.080	13.694
0.500	40.50	0.012	1.786	0.999	-0.022	13.896	0.058	2.688	0.862	-0.060	13.669
0.600	40.77	0.001	1.803	1.039	-0.018	13.507	0.041	2.638	0.916	-0.050	13.518
0.800	41.07	-0.010	1.807	1.082	-0.013	13.597	0.021	2.490	0.981	-0.038	13.365
1.000	41.17	-0.014	1.786	1.098	-0.010	13.109	0.011	2.356	1.012	-0.030	13.204
1.500	37.65	-0.032	1.656	1.168	0.004	10.443	-0.015	1.944	1.110	-0.013	11.910
2.000	29.10	-0.021	1.694	1.121	-0.004	8.935	-0.019	1.835	1.112	-0.005	9.524
3.000	22.19	-0.009	1.635	1.066	-0.009	12.745	-0.002	1.686	1.044	-0.013	12.148
4.000	20.37	0.007	1.573	1.010	-0.017	12.111	0.017	1.587	0.979	-0.025	12.293
5.000	19.57	0.014	1.508	0.984	-0.022	13.294	0.022	1.499	0.960	-0.034	13.912
6.000	19.11	0.023	1.463	0.958	-0.029	13.395	0.028	1.432	0.945	-0.035	12.803
8.000	18.62	0.033	1.381	0.934	-0.037	13.440	0.034	1.337	0.930	-0.033	12.173
10.000	18.40	0.039	1.319	0.922	-0.042	13.465	0.045	1.281	0.902	-0.047	13.902
15.000	18.24	0.066	1.238	0.861	-0.066	13.774	0.035	1.176	0.945	-0.038	14.496

Table S5. (EBF and EABF) G–P fitting coefficients (b, c, a, Xk and d) of AL10.0 glass sample.

Energy (MeV)	Z_{eq}	G-P Fitting Parameters for EBF					G-P Fitting Parameters for EABF				
		a	b	c	d	X_k	a	b	c	d	X_k
0.015	16.38	0.288	1.015	0.334	-0.259	14.249	0.270	1.015	0.334	-0.187	14.984
0.020	17.82	0.250	1.028	0.320	-0.208	19.651	0.161	1.024	0.451	-0.320	30.421
0.030	18.52	0.232	1.074	0.365	-0.131	12.917	0.248	1.075	0.348	-0.144	12.996
0.040	26.69	0.246	1.054	0.335	-0.113	12.320	0.236	1.053	0.346	-0.169	16.686
0.050	27.58	0.238	1.084	0.359	-0.138	13.404	0.243	1.087	0.349	-0.138	13.583
0.060	28.09	0.228	1.120	0.376	-0.129	13.725	0.244	1.134	0.349	-0.146	14.580
0.080	28.81	0.191	1.194	0.447	-0.109	14.000	0.215	1.252	0.398	-0.122	14.432
0.100	33.79	0.217	1.234	0.422	-0.118	13.832	0.214	1.304	0.421	-0.121	17.095
0.150	35.08	0.138	1.376	0.580	-0.073	14.211	0.249	1.774	0.394	-0.149	13.813
0.200	35.86	0.121	1.568	0.651	-0.072	14.004	0.233	2.418	0.462	-0.153	13.782
0.300	36.81	0.053	1.687	0.836	-0.035	13.784	0.116	2.578	0.688	-0.080	13.611
0.400	37.37	0.020	1.773	0.968	-0.027	13.322	0.073	2.715	0.826	-0.070	13.367
0.500	37.74	0.003	1.812	1.039	-0.020	13.057	0.042	2.672	0.925	-0.051	13.225
0.600	37.99	-0.007	1.821	1.076	-0.016	12.476	0.027	2.605	0.973	-0.043	12.943
0.800	38.25	-0.015	1.815	1.112	-0.013	12.256	0.010	2.445	1.028	-0.033	12.431
1.000	38.34	-0.018	1.792	1.120	-0.011	11.962	0.002	2.310	1.053	-0.026	11.968
1.500	34.52	-0.034	1.682	1.174	0.006	13.081	-0.018	1.947	1.123	-0.011	10.667
2.000	25.99	-0.021	1.712	1.123	-0.006	7.978	-0.026	1.827	1.135	0.007	25.383
3.000	20.16	-0.011	1.640	1.070	-0.007	13.202	-0.005	1.687	1.050	-0.010	11.965
4.000	18.70	0.008	1.579	1.003	-0.017	11.386	0.014	1.590	0.984	-0.023	12.074
5.000	18.07	0.015	1.513	0.979	-0.021	13.382	0.017	1.501	0.969	-0.029	15.451
6.000	17.69	0.019	1.462	0.965	-0.023	13.665	0.026	1.438	0.945	-0.032	13.351
8.000	17.30	0.030	1.383	0.934	-0.033	13.403	0.033	1.345	0.926	-0.031	12.553
10.000	17.12	0.037	1.323	0.919	-0.038	13.548	0.040	1.285	0.910	-0.041	13.705
15.000	16.99	0.060	1.236	0.869	-0.059	13.561	0.035	1.183	0.935	-0.038	14.388

Table S6. (EBF and EABF) G–P fitting coefficients (b, c, a, Xk and d) of AL12.5 glass sample.

Energy (MeV)	Z_{eq}	G-P Fitting Parameters for EBF					G-P Fitting Parameters for EABF				
		a	b	c	d	X_k	a	b	c	d	X_k
0.015	15.54	0.274	1.017	0.341	-0.196	11.151	0.261	1.017	0.341	-0.147	11.654
0.020	16.50	0.234	1.036	0.334	-0.333	25.524	0.234	1.035	0.345	-0.454	29.136
0.030	17.09	0.236	1.096	0.363	-0.135	13.645	0.236	1.096	0.363	-0.135	13.645
0.040	25.94	0.248	1.059	0.336	-0.119	11.672	0.248	1.059	0.331	-0.131	13.929
0.050	26.84	0.235	1.091	0.362	-0.137	13.683	0.243	1.095	0.347	-0.137	13.959
0.060	27.34	0.221	1.130	0.386	-0.124	13.880	0.239	1.146	0.357	-0.143	14.622
0.080	28.06	0.188	1.213	0.453	-0.106	14.124	0.209	1.274	0.409	-0.118	14.608
0.100	31.08	0.188	1.263	0.468	-0.104	13.864	0.169	1.346	0.486	-0.103	18.369
0.150	32.16	0.121	1.441	0.623	-0.065	14.110	0.230	1.932	0.430	-0.143	13.675
0.200	32.82	0.092	1.621	0.728	-0.057	13.844	0.186	2.495	0.549	-0.128	13.644
0.300	33.62	0.035	1.755	0.907	-0.029	13.300	0.087	2.710	0.777	-0.069	13.303
0.400	34.11	0.007	1.822	1.028	-0.022	12.651	0.048	2.744	0.914	-0.057	12.943
0.500	34.41	-0.008	1.846	1.092	-0.017	11.955	0.020	2.651	1.007	-0.040	12.640
0.600	34.61	-0.016	1.845	1.124	-0.013	11.114	0.008	2.562	1.048	-0.033	12.183
0.800	34.83	-0.023	1.826	1.150	-0.012	10.486	-0.004	2.386	1.089	-0.026	11.198
1.000	34.90	-0.023	1.800	1.148	-0.011	10.451	-0.011	2.249	1.106	-0.020	10.341
1.500	30.72	-0.036	1.716	1.181	0.009	16.615	-0.023	1.950	1.140	-0.009	9.002
2.000	22.59	-0.026	1.725	1.136	0.002	13.121	-0.028	1.831	1.140	0.007	20.995
3.000	18.13	-0.006	1.657	1.051	-0.009	11.575	-0.007	1.691	1.053	-0.006	12.838
4.000	17.04	0.008	1.583	1.000	-0.016	10.612	0.010	1.592	0.991	-0.017	12.806
5.000	16.57	0.014	1.514	0.979	-0.018	12.600	0.018	1.509	0.964	-0.026	14.949
6.000	16.28	0.020	1.467	0.958	-0.023	13.496	0.022	1.443	0.952	-0.030	14.842
8.000	15.98	0.031	1.388	0.927	-0.032	13.434	0.029	1.351	0.931	-0.026	13.070
10.000	15.85	0.035	1.326	0.917	-0.034	13.664	0.039	1.292	0.907	-0.037	13.469
15.000	15.75	0.049	1.230	0.893	-0.048	13.197	0.037	1.191	0.924	-0.038	14.300

Table S7. (EBF and EABF) G–P fitting coefficients (b, c, a, Xk and d) of AL15.0 glass sample.

Energy (MeV)	Z_{eq}	G-P Fitting Parameters for EBF					G-P Fitting Parameters for EABF				
		a	b	c	d	X_k	a	b	c	d	X_k
0.015	14.62	0.231	1.020	0.380	-0.147	11.999	0.204	1.020	0.404	-0.109	11.691
0.020	14.94	0.175	1.042	0.432	-0.079	14.058	0.193	1.042	0.412	-0.097	13.823
0.030	15.38	0.214	1.131	0.391	-0.115	14.208	0.217	1.131	0.389	-0.120	13.878
0.040	25.16	0.246	1.067	0.339	-0.121	11.952	0.245	1.067	0.337	-0.130	13.919
0.050	26.08	0.232	1.098	0.366	-0.136	13.980	0.243	1.104	0.344	-0.136	14.360
0.060	26.57	0.214	1.140	0.397	-0.118	14.045	0.233	1.158	0.366	-0.139	14.666
0.080	27.27	0.185	1.233	0.460	-0.103	14.257	0.202	1.299	0.421	-0.115	14.796
0.100	27.76	0.157	1.328	0.527	-0.087	13.978	0.151	1.469	0.517	-0.094	17.648
0.150	28.53	0.097	1.539	0.685	-0.054	14.010	0.199	2.165	0.489	-0.134	13.732
0.200	28.99	0.052	1.695	0.835	-0.036	13.619	0.120	2.604	0.671	-0.093	13.449
0.300	29.52	0.009	1.852	1.009	-0.021	12.606	0.045	2.900	0.903	-0.052	12.861
0.400	29.84	-0.014	1.893	1.116	-0.015	11.669	0.010	2.786	1.043	-0.037	12.322
0.500	30.03	-0.025	1.896	1.169	-0.012	10.337	-0.012	2.620	1.127	-0.023	11.781
0.600	30.15	-0.031	1.879	1.196	-0.010	9.100	-0.020	2.497	1.159	-0.019	11.058
0.800	30.27	-0.034	1.842	1.208	-0.012	7.838	-0.026	2.297	1.182	-0.016	9.353
1.000	30.30	-0.032	1.812	1.191	-0.011	8.178	-0.030	2.158	1.186	-0.012	7.892
1.500	25.78	-0.040	1.751	1.197	0.011	15.896	-0.039	1.938	1.194	0.010	15.358
2.000	18.99	-0.029	1.744	1.144	0.006	16.495	-0.030	1.835	1.147	0.007	16.451
3.000	16.07	-0.009	1.660	1.059	-0.006	10.261	-0.010	1.691	1.059	-0.002	11.917
4.000	15.39	0.007	1.589	1.000	-0.016	13.124	0.008	1.594	0.996	-0.014	12.813
5.000	15.09	0.016	1.520	0.969	-0.017	12.015	0.022	1.523	0.949	-0.028	14.542
6.000	14.88	0.018	1.470	0.957	-0.019	14.276	0.022	1.452	0.946	-0.029	15.545
8.000	14.69	0.028	1.389	0.929	-0.027	13.609	0.036	1.363	0.910	-0.031	11.804
10.000	14.60	0.037	1.330	0.907	-0.034	13.348	0.037	1.298	0.908	-0.033	13.562
15.000	14.53	0.050	1.235	0.883	-0.047	13.211	0.035	1.197	0.922	-0.035	14.408
